# Silicon Nitride Photonic Integration Platforms for Visible, Near-Infrared and Mid-Infrared Applications

**DOI:** 10.3390/s17092088

**Published:** 2017-09-12

**Authors:** Pascual Muñoz, Gloria Micó, Luis A. Bru, Daniel Pastor, Daniel Pérez, José David Doménech, Juan Fernández, Rocío Baños, Bernardo Gargallo, Rubén Alemany, Ana M. Sánchez, Josep M. Cirera, Roser Mas, Carlos Domínguez

**Affiliations:** 1 Photonics Research Labs, Universitat Politècnica de València, c/ Camino de Vera s/n, 46021 Valencia, Spain; glomica@iteam.upv.es (G.M.); luibror@doctor.upv.es (L.A.B.); dpastor@dcom.upv.es (D.P.); dapelo2@teleco.upv.es (D.P.); ralemany@upvnet.upv.es (R.A.); 2 R&D Department, VLC Photonics S.L., c/ Camino de Vera s/n, 46021 Valencia, Spain; david.domenech@vlcphotonics.com (J.D.D.); juan.fernandez@vlcphotonics.com (J.F.); rocio.banos@vlcphotonics.com (R.B.); bernardo.gargallo@vlcphotonics.com (B.G.); 3 Grupo de Transductores Químicos (GTQ), Instituto de Microelectrónica de Barcelona (IMB-CNM, CSIC), 08193 Cerdanyola del Vallès, Barcelona, Spain; ana.sanchez@imb-cnm.csic.es (A.M.S.); JosepMaria.Cirera@imb-cnm.csic.es (J.M.C.); roser.mas@imb-cnm.csic.es (R.M.); Carlos.Dominguez@imb-cnm.csic.es (C.D.)

**Keywords:** silicon nitride photonics, propagation loss, group index, group velocity dispersion, birefringence, full-field optical measurements, silicon photonics, photonic integrated circuits, generic integration, multi-project wafer

## Abstract

Silicon nitride photonics is on the rise owing to the broadband nature of the material, allowing applications of biophotonics, tele/datacom, optical signal processing and sensing, from visible, through near to mid-infrared wavelengths. In this paper, a review of the state of the art of silicon nitride strip waveguide platforms is provided, alongside the experimental results on the development of a versatile 300 nm guiding film height silicon nitride platform.

## 1. Introduction

**Figure 1:**
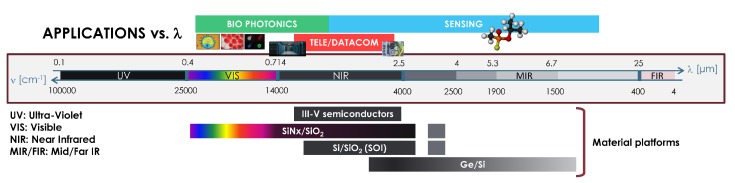
Applications versus wavelength range, and the different material systems, III–V semiconductor and Silicon photonics, commonly employed in generic photonic integration (Reference [3], adapted with permission from OSA Publishing).

**Figure 2:**
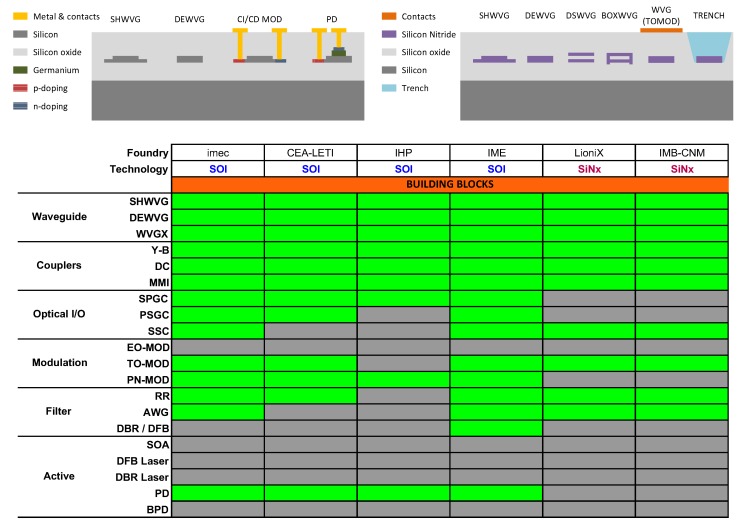
Silicon photonics platform cross-sections (top) and building blocks per foundry (bottom). Color code: Green = Available/Possible, Grey = Not Available/Possible. Abbreviations: SHWVG Shallow waveguide, DEWVG Deeply etched waveguide, DSWVG double-stripe waveguide, WVGX Waveguide crossing, Y-B Y-branch, DC Directional coupler, MMI Multi-Mode Interference coupler, SPGC Single Polarization Grating Coupler, PSGC Polarization Splitting GC, SSC Spot-Size Converter, EO-MOD Electro-Optic Modulator, TO-MOD Thermo-Optic Modulator, PN-MOD PN Junction Modulator, RR Ring Resonator, AWG Arrayed Waveguide Grating, DBR Distributed Bragg Reflector, SOA Semiconductor Optical Amplifier, PD Photo-Detector, BPD Balanced PD.

Despite many materials being amenable to producing photonic integrated circuits (PICs), only a few have been employed to mirror the path of the semiconductors electronics industry and evolved into an eco-system of foundries, software suppliers, design houses and fabless companies [1,2]. The material systems for which generic processes and offering access through multi-project wafer (MPW) runs [3] have been developed are Silicon on Insulator (SOI) [4], Indium Phosphide [5] and Silicon Nitride [6]. Applications are tightly matted to wavelength, which determines the required transparency range for the materials (semiconductors, dielectrics) to be employed. In Figure 1, the optical spectrum is sketched alongside the wavelength ranges for main applications, from shorter to longer wavelengths, biophotonics, tele/datacom, and sensing, from visible, through near to mid-infrared. III-V semiconductors are mainly used in the near infrared (NIR), while SOI has slightly broader wavelength range. On the other hand, silicon nitride on silicon dioxide platforms are of use from visible (VIS) wavelengths to the upper part of the NIR (as SOI). The main limitation for SOI and Si${}_{3}$N${}_{4}$ platforms in the upper part of the spectrum is the strong absorption of SiO${}_{2}$ above approximately 4 μm. Hence, other material combinations such as germanium on silicon platforms have been proposed [7,8,9,10,11]. Silicon based opto-electronics covers the aforementioned wavelength ranges and applications. A sketch of the common cross-sections employed in SOI and Si${}_{3}$N${}_{4}$ is shown in the upper part of Figure 2. The different foundries supplying silicon based generic technologies through MPW runs are given in the table within the same figure.

Silicon on Insulator is a semiconductor technology where components are etched/patterned/fabricated in a 180–220 nm Si layer placed on top of a 1–3 μm insulator [4]. Si passives are formed by initial few mask layers through partial and/or full Si etching steps after which multiple ion implantations are conducted for active devices, such as Ge photodetectors and Si modulators. Coupling into and outside of the chip can be performed via edge couplers (with typical losses of 1dB/facet) or vertically, via Si surface gratings (2 dB/coupler with 40–70 nm 3-dB bandwidth). The main advantage of SOI technology resides in its compatibility with CMOS fabrication processes and the infrastructure used in microelectronics and thus the compatibility in terms of System on Chip (SoC), System in Package (SiP), reproducibility and cost. Refractive index contrast is over 100% (*n* = 3.4 for Si and *n* = 1.45 for SiO${}_{2}$), leading to small footprint circuits. Two main types of waveguides are available, as shown on the top-left panel of Figure 2: rib waveguides (1–8 μm width), which exhibit relatively low losses down to 0.1–0.5 dB/cm, but are limited in bending radius to around 100 μm, and strip waveguides (500 nm width) which exhibit much higher losses (1–3 dB/cm) but support lower values for minimum bending radius (5–20 μm). Integration density on a chip is currently exceeding 4000 components, and the component count integration trend is exceeding the rate given by Moore’s law indeed. Several building blocks are available in monolithic SOI, including: passives, such as arrayed waveguide gratings and optical filters, and actives as Ge photodetectors, ring and travelling-wave electro-refractive modulators (up to 50 GHz). A table of the available building blocks for the different foundries supplying generic processes through multi-project wafer (MPW) runs is also given at the bottom of Figure 2. The main disadvantages of monolithic SOI technology is that it does not support optical sources and optical amplifiers, and the Pockels effect is poor, so no electro-optic efficient modulators are possible. To overcome this limitation III-V functionalities have to be integrated into the SOI platform by means of either molecular or adhesive wafer bonding [12].

Dielectric based photonic technology started with the development of components in the visible wavelength range, applied to build optical sensors [13]. This waveguide technology is based on a combination of (stoichiometric) silicon nitride (SiN${}_{x}$) Si${}_{3}$N${}_{4}$ as waveguide layers, filled by and encapsulated with silica (SiO${}_{2}$) as cladding layers on a silicon wafer. SiO${}_{2}$ and Si${}_{3}$N${}_{4}$ layers are fabricated with CMOS-compatible techniques like thermal oxidation and industrial standard low-pressure LPCVD) and plasma enhanced (PECVD) chemical vapour deposition techniques equipment that enables cost-effective volume production. Several waveguide cross-sectional geometries are available, top-right panel in Figure 2. In general the cross-sections shown perform at 1550 nm with losses below 0.5 dB/cm and minimum bending radius typically around 150 μm. In/out-coupling is achieved by means of adiabatically tapered spot-size converters with <1 dB coupling loss to standard single mode fiber. Several fundamental building blocks are available including the optical waveguide, thermo-optic phase tuning elements, directional and Multi-Mode Interference couplers, as detailed in Figure 2 as well. From these more complex subsystems have been demonstrated. The main disadvantage of this technology is that no optical sources, detectors, amplifiers and modulators are available in the generic MPW processes. However, the operation wavelength range spans from visible to the mid infrared, with very low loss. The integration with these active building blocks requires a hybrid or heterogeneous approach with separately fabricated InP or Silicon platform chips. In summary, whereas SOI provides all the active building blocks (except the optical source), such as waveguides and waveguide based blocks, modulators and detectors, up to the date silicon nitride generic MPW processes offer only passive optical waveguides, and just thermo-optic tuners as phase shifters.

In this paper, we report on our progress on a moderate confinement Si${}_{3}$N${}_{4}$/SiO${}_{2}$ waveguide platform amenable for biophotonics, tele/datacom and sensing applications from the VIS to the long NIR (400–3700 nm wavelength range). The paper is structured as follows. Section 2 summarizes the present state of the art of strip waveguide platforms using silicon nitride and compounds on silicon oxide. In Section 3 we report our developments on the Si${}_{3}$N${}_{4}$/SiO${}_{2}$ platform with guiding film height of 300 nm. Advanced interferometric full-field characterization techniques are employed with suitable on-chip test structures, in order to gather the platform waveguiding figures of merit including: propagation loss, group index, group velocity dispersion (GVD) and birefringence. Complementary modeling aspects are given in the appendix. The characterization of thermo-optic phase shifters and fiber in/out coupling structures is also reported. In Section 4 we present means for extending the operational wavelength range for Si${}_{3}$N${}_{4}$ based platforms and the conclusion is given in Section 5.

## 2. Silicon Nitride Photonic Integration Platforms: State of the Art

Si${}_{3}$N${}_{4}$ material is widely used in the fabrication of microelectronic circuits, as a support material for developing the devices with other compounds, with whom it exhibits tight electronic, structural and chemical interrelations [14]. For photonics, Stulius and Streifer reported in 1977 [15] the first fabrication of Si${}_{3}$N${}_{4}$ films on a SiO${}_{2}$ buffer on Silicon wafers, for light propagation in the red visible wavelength (632 nm). After some works in the 1980s on the propagation of visible (VIS) light through straight channel Si${}_{3}$N${}_{4}$ waveguides, a seminal contribution on the application of this material in a functional device was done by Heideman E.A. [16], with a partially integrated Mach-Zehnder Interferometer (MZI) for immunosening assays, where the two arms of the MZI were in fact Si${}_{3}$N${}_{4}$/SiO${}_{2}$ waveguides, while the optical couplers for the MZI were external to the photonic chip.

Despite a fully integrated MZI sensor was reported a few years latter [17] in the late 1990s, new interests on this material platform started again back in 2005, when Sandia Labs (USA) [18] and Univ. Trento (ITA) [19] developed processes and demonstrated applications in the near infrared (NIR). They were followed with silicon oxynitride (SiONx) waveguides [20] and Si${}_{3}$N${}_{4}$ waveguides [21,22,23] from 2008 to 2011. Up to 2011 demonstrations by telecom related groups are for NIR C-Band at 1550 nm, and all the waveguide cross-sections were for moderate confinement (film heights > 100 nm), despite some groups reported by 2011 low confinement waveguides (film h < 100 nm) [22]. By 2013, researchers [24,25] set new paths of Si${}_{3}$N${}_{4}$ technology for VIS applications. In parallel, and since 2011, there is a growing interest on high confinement (film h > 400 nm) waveguides for the long NIR (NIR+) (wavelength > 2000 nm), which are reported by several groups [26,27,28,29]. In 2015–2016, new players in moderate confinement technologies appear [30,31,32].

A summary of the reported strip waveguide silicon nitride platforms is given in Table 1. The table collates information on the operation wavelength, layer stack, cross-section dimensions, and when available, cut-off wavelength, propation loss and bend radius. Mechanisms responsible for optical propagation loss in strip silicon nitride waveguides have been previously described and experimentally explored, employing different fabrication recipes, by Sandia Labs in [18]. In short, provided processes are put into place to remove impurities in the silicon nitride and silicon oxide layers (e.g., through annealing), the surface roughness (film roughness and waveguide sidewall roughness) together with the mode confinement at the operation wavelength (given by the waveguide cross-section, width, height, as well by the substrate and cladding heights) are the main factors determining the propagation loss. Therefore, those cross-sections for which the optical mode feels less side-wall roughness (either because of strong confinement, low roughness or both), will be prone to lower propagation loss. In what follows, the state of the art for strip waveguides presented in Table 1 is discussed, grouping the platforms by wavelength range and optical confinement. Other types of waveguides, such as box waveguide and double strip waveguide (Figure 2, top-right, BOXWVG and DSWVG respectively), have been reported [33,34,35] with propagation loss and bend radius as low as 0.1 dB/cm and 70 μm.

For use in the NIR, low confinement strip waveguides were demonstrated together by LioniX and UCSB, with guiding film heights ∼100 nm, waveguide widths ∼2800 nm and propagation loss as low as 0.09 dB/cm @ 1550 nm for 0.5 mm bend radius. The lowest loss reported by these groups is 0.001 dB/cm. The low propagation loss is due to the low confinement in the Si${}_{3}$N${}_{4}$, being most of the mode guided through the SiO${}_{2}$, enabled by huge layers of the buried oxide (BOX) and cladding (8 μm and 7.5 μm respectively). Still for the NIR, moderate confinement waveguides (nitride height in between 150–400 nm) have been demonstrated by several groups. Sandia (2005) [18] and UCD (2015) [31] reported LPCVD Si${}_{3}$N${}_{4}$ guiding film heights of ∼150–200 nm, with waveguide widths ∼800–2000 nm. The propagation loss reported is 0.11–1.45 dB/cm@1550 nm for BOX height up to 5.0 μm. Other groups as IME and University Toronto have reported 3D SiNx on top of SOI in the NIR [36], employing LPCVD Si${}_{3}$N${}_{4}$ guiding film heights ∼300–400 nm, with waveguide widths ∼800–1000 nm, resulting into propagation loss of 1.30–2.10 dB/cm @ 1550 nm for BOX heights in between 2.0 μm and 5.0 μm. Using similar film heights in the VIS and VIS+ wavelength ranges, University Aachen and University Gent reported PECVD guiding film heights ∼100–220 nm, waveguide widths ∼300–800 nm PECVD guiding film loss 0.51–2.25 dB/cm @ 532–600 nm for BOX height ∼2400 nm.

Finally, both for the NIR and NIR+ wavelength ranges, high confinement waveguides have been reported by Kippengerg (EPFL) [26], Lipson (Cornell, then Columbia) [27,29] and Agarwal (MIT) in 2013 [37], followed in 2015 by Torres (Chalmers) [28] and companies such as LioniX [38] and LigenTec [39] (EPFL spin-off). Guiding film heights ∼700–2500 nm, with waveguide widths ∼700–4000 nm and propagation loss of 0.04–1.37 dB/cm @ 1550 nm and 0.16–2.1 dB/cm @ 2600–3700 nm, for BOX heights in the range of 2.0–8.0 μm have been reported.

## 3. Silicon Nitride Platform With 300 nm Film Height

The state of the art compiled in Table 1 is summarized in Table 2 in terms of the confinement and wavelength range. Cross-section dimensions determine the guiding properties of waveguides: number of modes, polarization dependence, confinement (loss), dispersion profile and non-linear propagation coefficient. Our goal was to have a versatile platform, covering the widest wavelength range as possible with two polarizations in the fundamental mode.

**Figure 3:**
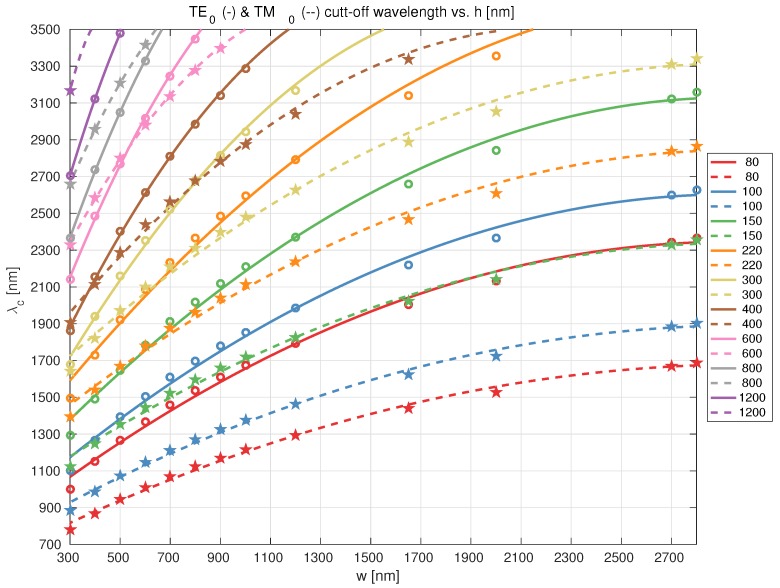
Strip silicon nitride waveguide cut-off wavelength for TE${}_{0}$ and TM${}_{0}$ modes vs. waveguide width, and for different Si${}_{3}$N${}_{4}$ film heights (80–1200 nm) (symbols: simulation points; lines: fit, continuous TE${}_{0}$, dashed TM${}_{0}$.

Firstly, through simulation (see appendix for details) we firstly determined the cut-off wavelength for the first order mode and the two polarizations, for strip waveguides with guiding film height and waveguide width ranges of 80–1200 nm and 300–2750 nm respectively. These cover most of the state of the art of Table 1. The results are shown in Figure 3. At the sight of graph and the TM${}_{0}$ mode, previously reported platforms with film heights 80–220 nm were discarded, since at most this mode propagates up to λ≃2.9 μm for h = 220 nm.

Conversely, platforms reported with film heights of 400 nm and above are mainly used for non-linear optical signal processing, such as frequency comb and super-continuum generation [27,43,44,45]. However, non-linear applications are non exclusive of high confinement in the NIR and NIR+, since they have also been reported in the VIS+ range (cf. [46]). Whereas the former make use of thicker nitride guiding layers, the latter can be achieved with film heights in the range of 100–400 nm. From a fabrication point of view, these heights can be obtained in a single deposition step, with reduced risk of nitride cracking due to stress issues (see for instance [29]).

On other hand, when the goal is to minimize propagation loss, low or high confinement waveguides are considered. Confinement is related to propagation loss due to the interaction of the propagating mode with sidewall roughness [18]. Both low and high confinement minimize the effect of wall roughness. Whereas low confinement platforms are strongly polarization dependent (i.e., only one polarization is guided as for instance in [23]), high confinement ones suffer of multi-modal effects-lateral and vertical—(cf. [47]). Low confinement waveguides are usually employed in linear applications, such as optical delay lines, whereas high confinement waveguides aim at having the lowest non-linear effects threshold as possible.

In summary, the existing low and high confinement Si${}_{3}$N${}_{4}$ platforms are best suited for specific applications, whereas moderate confinement platforms are versatile, at the cost of reduced performance (loss, non-linear threshold). Nonetheless, the latter can also be tweaked to tailor specific performance metrics, as it will be outlined in Section 4.

Owing to all the above, and aiming at covering a wavelength range from the VIS to the long NIR (400–3700 nm wavelength range) for photonic integrated applications such as biophotonics, tele/datacom and sensing, we developed a Si${}_{3}$N${}_{4}$ on SiO${}_{2}$ platform with Si${}_{3}$N${}_{4}$ guiding film height of 300 nm. In this section, details on the fabrications processes and resulting linear operation performance in the optical telecom C-band (1550 nm wavelength range) for which the lab equipment was readily available, are reported. Characterization in the VIS and NIR+ will follow in subsequent papers. Modeling aspects for linear and non-linear propagation figures of merit are provided in the appendix.

### 3.1. Fabrication Process

The fabrication process makes use of 100 mm (4 inch) Si wafers. A layer stack of SiO${}_{2}$/Si${}_{3}$N${}_{4}$/SiO${}_{2}$ is formed on top of the wafer as follows. Firstly, a SiO${}_{2}$ buffer (2.5 μm thick, *n* = 1.464) is grown by thermal oxidation of the silicon substrate. Following a Si${}_{3}$N${}_{4}$ layer is deposited via Low Pressure Chemical Vapor Deposition (LPCVD), with thickness 300 nm (*n* = 2.01). The fabrication process parameters of the bilayer are selected in order to maintain the substrate as flat as possible, reducing the mechanical stress of the bilayer on the substrate. Two different waveguide structures are defined by photo-lithography with an i-line stepper (minimum feature size 500 nm) followed by a reactive ion etching (RIE) of the silicon nitride film. The 300 nm silicon nitride layer may be etched completely to form a strip waveguide structure (DEWVG in Figure 2), or the etching is done partially obtaining a rib waveguide structure (SHWVG, Figure 2) 300 nm/150 nm. By properly combining the mask layers on the design stage, a strip waveguide of just 150 nm height can be defined as well. Finally, a 2.0 μm thick SiO${}_{2}$ (*n* = 1.45) is deposited by Plasma Enhanced CVD (PECVD) to complete the different waveguiding cross-sections. In addition to the waveguides, two additional processes allow for defining the thermo-optic tuners (heaters) and selective area trenching (air wells). A metal heater (TOMOD, Figure 2) is obtained by sequential evaporation of 30 nm Chromium and 100 nm Gold, and defined by a lift-off process. The air wells (TRENCH) are opened into the SiO${}_{2}$ cladding layer down to the bottom of the silicon nitride guiding layer by means of photo-lithography followed by a RIE step. The TRENCH openings are the interfaces between the photonic elements and the surrounding media, when applied for (bio)chemical sensors [48].

### 3.2. Measurement Setup

**Figure 4:**
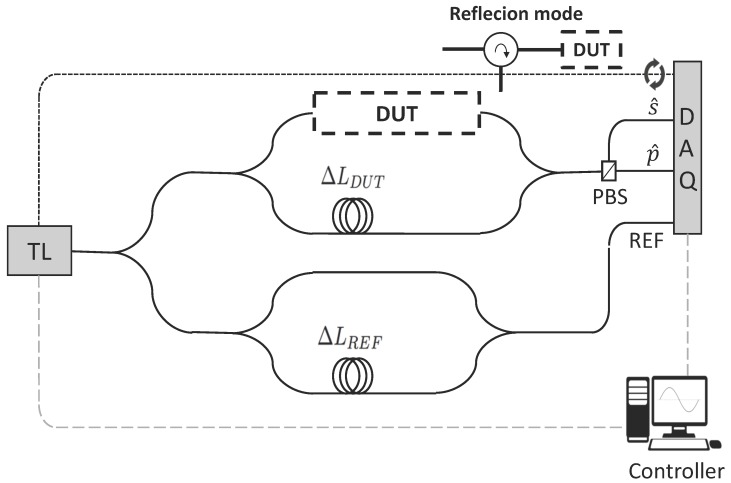
Optical frequency domain reflectometry setup. Abbreviations: Device under test (DUT), Reference (REF), polarization beam splitter (PBS).

The Optical Frequency Domain Reflectometry (OFDR) setup employed [49,50,51] for the measurements was composed of imbalanced MZIs in standard single-mode fiber, fed by a scanning Tunable Laser (TL), Figure 4. The upper MZI includes the device under test (DUT), in our case the Si${}_{3}$N${}_{4}$ chip in/out coupled with lensed fibers. The output lensed fiber is connected to a Polarization Beam Splitter (PBS), so two different interferograms are captured with two photo-detectors and registered through a digital acquisition (DAQ) card. This disposition prevents the destructive interfering effects due to polarization missalignment between both MZI arms, as described in [50,51]. The lower MZI provides the reference (or triggering) signal for the corrections of the TL wavelength sweeping phase error. As described in [49,50,51], the time responses of the DUT can be isolated after performing the Fast Fourier Transform (FFT) of the interferogramsD. When the setup is used in reflection mode, i.e., to determine waveguide propagation loss, a circulator is inserted between the DUT and the upper MZI arm. To check our measurement setup was working properly, we measured the full field transfer response of Arrayed Waveguide Gratings, and compared it with the classic transmission measurement. Further details on this and the OFDR setup can be found in [52].

### 3.3. Propagation Loss

**Figure 5:**
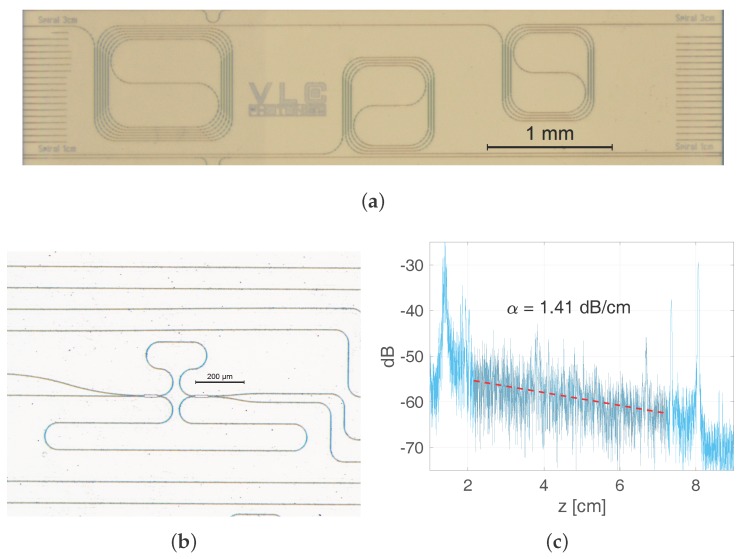
Test structures devised for the characterization of the propagation loss, (**a**) spiral waveguides and (**b**) Mach-Zehnder Interferometers and (**c**) optical frequency domain reflectometry trace from a spiral waveguide, the light blue trace is the measurement, the gray shaded part corresponds to the range selected for a linear fit, which is shown in the figure as dashed red line.

The propagation loss was derived both from the backscattering of spiral waveguides (width 1.0 μm) obtained via OFDR and the transmission spectrum of MZIs [53]. The spiral waveguide test structures are shown in the top panel of Figure 5, comprising lengths of 1 cm (bottom) and 1 + 2 cm (top). The bend radius employed in the spiral waveguides was 150 μm for negligible bend loss as per full-vectorial mode solver simulations. The OFDR acquired measurement is shown within the same figure, where the in/out coupling events can be clearly identified as the high peaks at the beginning and end of the recorded trace. Over the trace, the range selected to perform a linear fit is graded on gray color. The measurement was repeated for several samples manufactured with the same process steps, with very similar results.

To cross-check the propagation loss value obtained through reflectometry, MZI test structures were also included in the designs. The MZI layout was devised so as to have the length difference only in the straight sections of the width of interest, with a bend radius of 50 μm to reduce the footprint. The couplers employed in the MZI layout are 2 × 2 MMI couplers with even splitting ratio. A microscope picture of the MZI test structure is shown in Figure 5. Following the procedure described in [53], the four transmission spectra for the MZI were acquired by using a broadband Amplified Spontaneous Emission (ASE) source and an Optical Spectrum Analyzer (OSA). The propagation loss derived was in the range of 1.2–1.6 dB/cm.

Note in all the cases our devices were not subject to Si${}_{3}$N${}_{4}$ annealing. The propagation loss is hence in agreement to similar waveguide cross-sections, cf. [21,36] and Table 1, so further propagation loss reduction in the optical telecom C-band can be expected for annealed Si${}_{3}$N${}_{4}$ films (cf. [18]).

### 3.4. Group Index, Dispersion and Birefringence

**Figure 6:**
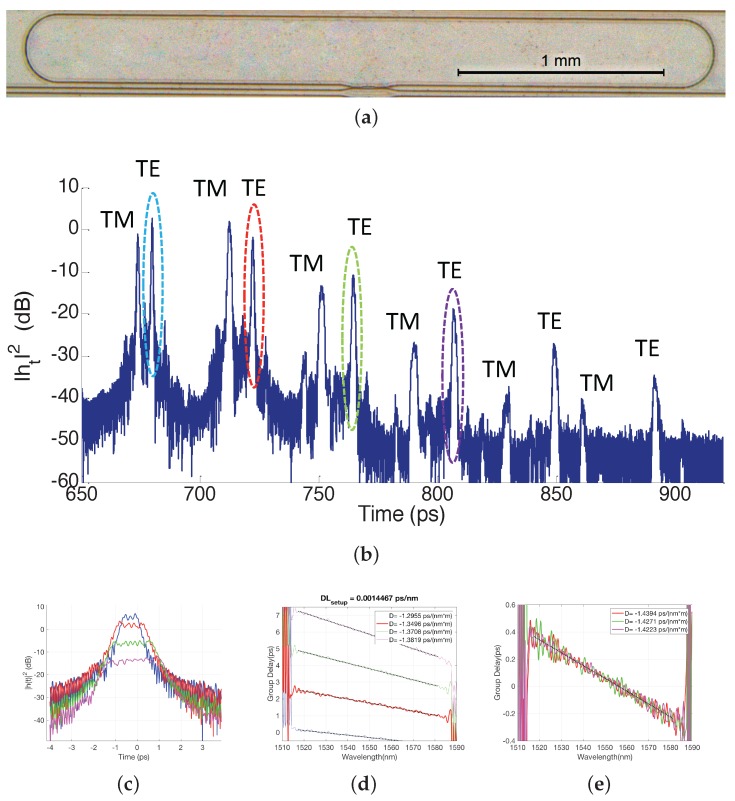
(**a**) Ring resonator test structure devised for the characterization of the group velocity dispersion and birefringence, (**b**) Optical Frequency Domain Reflectometry (OFDR) trace from the ring resonator, with TE and TM pulses labeled, (**c**) TE pulses sliced and collated, exhibiting broadening due to group velocity dispersion (GVD), and (**d**,**e**) their corresponding group delay.

In order to measure group index, dispersion and birefringence, ring resonator (RR) coupled with a 2 × 2 MMI to a straight waveguide was devised as test structure, where the MMI design was for even splitting, and the ring radius of the bent sections in the ring perimeter was 150 μm, which as per our full-vectorial mode solver provides similar guiding characteristics (effective index versus wavelength) as straight waveguides of the same width (1.0 μm). The RR total perimeter was 6.63 mm. A microscope picture of the fabricated device is shown in Figure 6a.

In the OFDR setup, the TL scanning speed was 40 nm/s with a 80 nm span (centered at 1555 nm). After performing the FFT of the interferograms, the power time response in Figure 6b was obtained. The different peaks in the trace correspond to multiple recirculations from the RR. Furthermore, the TE and TM splitting in time can also be observed. From the relative positions of the peaks in the trace and the dimensions of the ring, group indices for TE and TM were determined to be 1.892 and 1.717 respectively. For TE, a group index in the range of 1.90–1.92 was inferred from MZI spectra transmission measurements, which is in good agreement to that obtained through OFDR. The TE and TM propagation delay difference leads to a birefringence value of 0.168.

Next, by isolating several consecutive pulses, i.e., slicing the trace around the TE pulses and representing them centered at the same time, a broadening effect can be clearly observed, due to GVD, Figure 6c. Each truncated response, for which amplitude and phase information is present thanks to the OFDR measurement method, is transformed into the frequency domain to calculate the group delay [50,51], and linearly fitted between 1514 nm and 1594 nm to obtain the dispersion parameters D (ps/(nm m)), as shown in Figure 6d.

**Figure A1:**
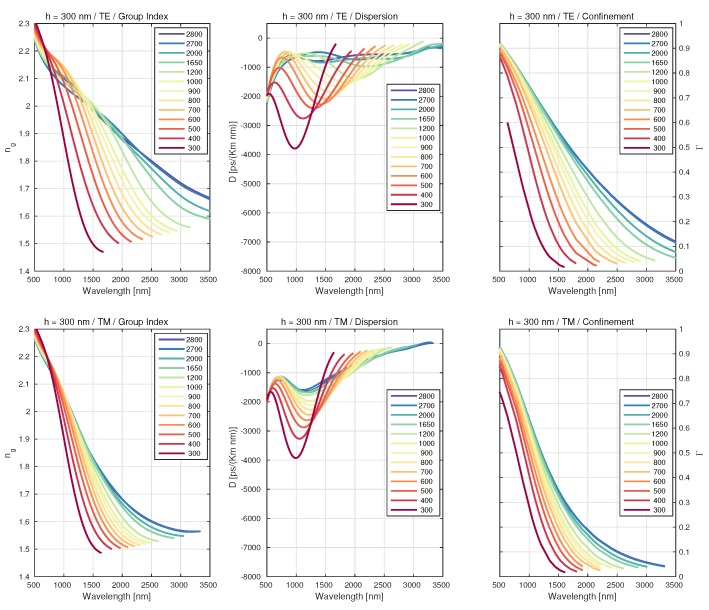
Linear propagation properties: group index, dispersion and confinement, vs. wavelength, for different waveguide widths.

**Figure A2:**
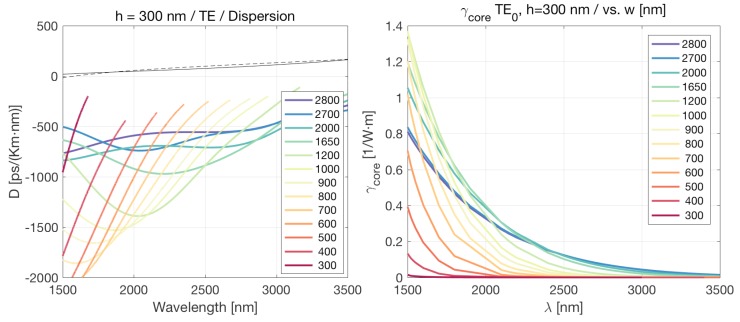
Enlarged view of the dispersion parameter, and non-linear propagation parameter, vs. wavelength, for different waveguide widths, for TE${}_{0}$.

The obtained D values were not constant as should be expected, suggesting the measurement setup dispersion is added to that from the chip test structure. However, the multiple recirculations from the RR can be related in pairs to isolate a single round trip pass along the ring. Hence, Figure 6e shows the group delay difference between adjacent TE pulses, alongside with a linear fit and the estimated dispersion calculated over the wavelength range of interest. This results into an average dispersion of D = −1.43 ps/(nm m) with a relative error of ±1.5%. Note the measured value is in good agreement with the modeled waveguides, as described in the Appendix, Figure A1 and Figure A2. From these values it is straightforward to obtain the dispersion offset from the set-up as ${\left(DL\right)}_{setup}=0.0014467$ ps/nm.

### 3.5. Thermo-Optic Phase Shifters

Aiming at the reconfiguration of PICs, various physical mechanisms exist and are present in the different technology platforms. Electro-absorption and electro-refraction are faster and use less energy, however the thermo-optic effect over the refractive index is larger, and induces less losses [54]. Despite their main drawbacks, power consumption and thermal cross-talk, and the proposed improvement alternatives [55,56] resorting to additional process steps, regular thermal tuners are simple and commonplace. However, most of the approaches in the literature, for all technologies, propose the use of long and wide tuners for linear phase-shift vs. driving power operation: in [57], a heater length of 2 mm is given, in [58], a 40 mm length tuner is presented and in [59], lengths above 600 μm are employed, to cite a few. In terms of the length, long heaters are contrary to the spirit of PICs, where footprint ultimately determines the cost of the photonic circuit. Furthermore, a common given figure given in the literature is the switching power to obtain a phase shift of π, namely $P_{\pi }$. For instance, in [58] with a technology similar to one being in this paper, a switching power of 350 mW, corresponding to a temperature increase of 40 ${}^{\circ }$C is reported.

**Figure 7:**
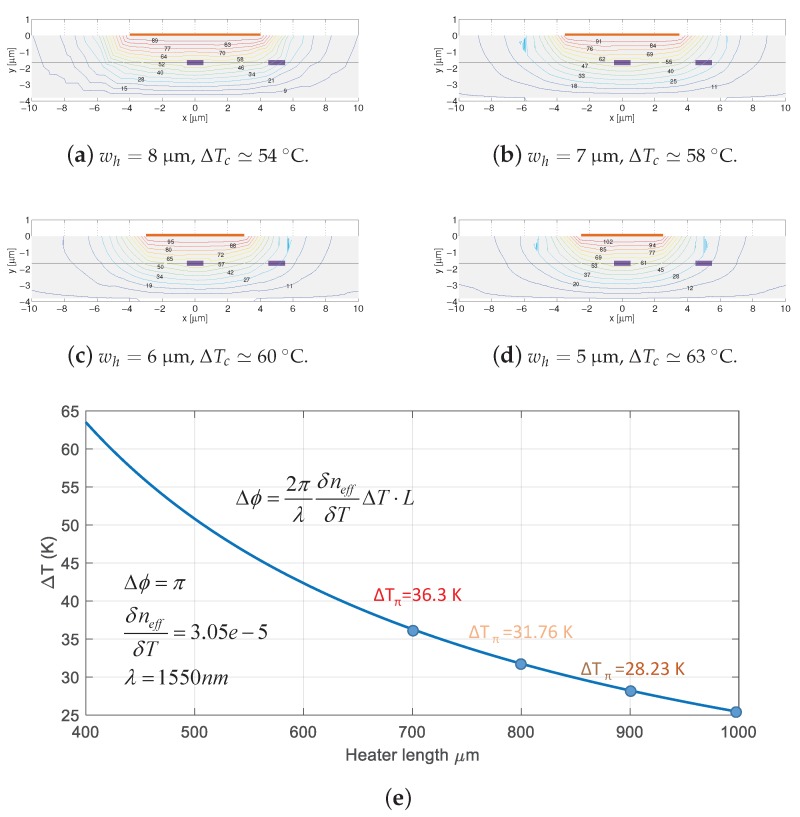
Waveguide cross-section with heater on top, and adjacent waveguide at a distance of 5 μm, with temperature gradient distribution overlaid. A dotted line is drawn from left to right crossing the core of the waveguides at half their height. Four different heater widths are shown (**a**–**d**), for the same heater length ($L_{h}=270$ μm). The contours are simulated for the same heater power consumption (same amount of heat generated), showing the temperature gradient is larger for narrow heaters. Panel (**e**) shows simulation results of temperature required for a π phase shift vs. heater length. Abbreviations employed in the figure: $w_{h}$ heater width; Δϕ, optical phase change; $\Delta T_{c}$ temperature change in the waveguide core; $\delta n_{eff}$ change in the effective index; $\Delta T_{\pi }$ temperature change causing a phase change of π.

The heater dimensions influence on the temperature gradient that is created in the waveguide cross-section. For the same amount of heat, that is for the same amount of electric consumed power at the heater, *P*, different temperature gradients result from heaters of different widths and lengths [60]. In Figure 7 four heaters with the same length, $L_{h}=270$ μm, and widths of 5, 6, 7 and 8 μm, are simulated for the same consumed power, with COMSOL MP software package. The silicon nitride waveguide, 1.0 × 0.3 μm${}^{2}$ sits on top of 2 μm of buried oxide, which in turn was grown on top of a silicon wafer of thickness 500 μm. In the simulations, the temperature at the bottom part of the silicon is fixed to 25 ${}^{\circ }$C. The voltage is set in the simulator for the wider heater ($w_{h}=8$ μm) to 2.5 V, for a previously determined resistance of ≃21 Ω (P=297.62 mW) (resistance measurements can be readily found in Figure 4 and Figure 5 on reference [60]). For the narrow heaters, *V* is set to provide the same power by using the resistance values from Figure 7. The resulting temperature gradient in the core of the silicon nitride waveguide is approximately 11 ${}^{\circ }$C more for the heater with narrower width. Hence, the same amount of heat is concentrated by narrow heaters, creating larger temperature gradients. Finally, Figure 7e shows the simulation results of the temperature required for a π phase shift vs. heater length. The waveguide core temperature with the SiO${}_{2}$ cladding height of our platform is approximately a 58% that of the heater. As expected, shorter heaters require a larger temperature gradient to obtain the same phase shift. Therefore, there is a trade-off between heater footprint and resilience/durability (larger temperature gradients can damage the metal stack in the heater). The electrical power required to obtain a given temperature gradient in the core, is linked to the construction parameters of the heater, metal stack and width [60].

**Figure 8:**
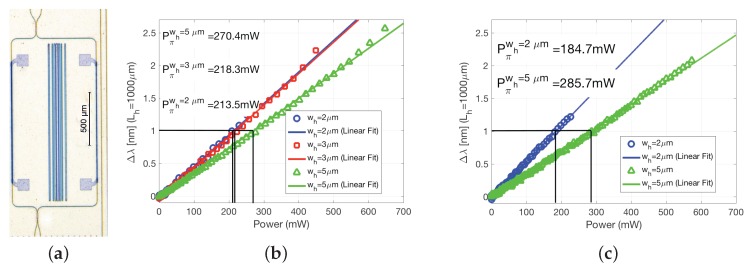
(**a**) Mach-Zehnder Interferometer (MZI) test structure for thermal-tuners, performance of tuners based on a metal layer stack of (**b**) 100 nm Au and 30 nm Cr and (**c**) 30 nm Au, 15 nm Ni, 10 nm Ti. Abbreviations: Δλ is the wavelength shift in the MZI spectrum due to the actuation of the heater; $P_{\pi }^{w_{h}}$ is the π shift power for a given heater width, $w_{h}$.

We investigated different heater configurations, different widths for a length of 1 mm, as well as different metal stacks, employing as test structures MZIs such as the one shown in Figure 8a. The MZIs were designed with a free spectral range (FSR) of 2 nm at 1550 nm. Hence, a π phase shift corresponds to 1 nm of wavelength shift in the spectral domain.

In Figure 8b, the performance of the thermal-tuners fabricate on a 100 nm Au and 30 nm Cr metal stack is shown, whereas Figure 8c the results are for heaters with a metal stack of 30 nm Au, 15 nm Ni and 10 nm Ti. Firstly, all the heaters with narrower metal width are more efficient (less power required for a π phase shift). This is consistent with the temperature gradient maps obtained through simulation, previously shown in Figure 7. Secondly, the maximum achievable phase shift does also depend on the heater width. By closes examining Figure 8b,c (the symbols, that correspond to actual measured values), one can notice how wider heaters can be operated to obtain larger phase shift range. This is due to the fact the heater gets damaged earlier for larger temperature gradients, which in turn occur for narrower heaters. Despite this cannot be inferred by comparing Figure 8b,c, in our experiments most of the heaters in Au/Cr (b) were damaged above certain operation power (temperature), whereas the ones with the Au/Ni/Ti metal stack (c) showed increased resiliency and durability.

### 3.6. Fiber in/out Coupling Structures

**Figure 9:**
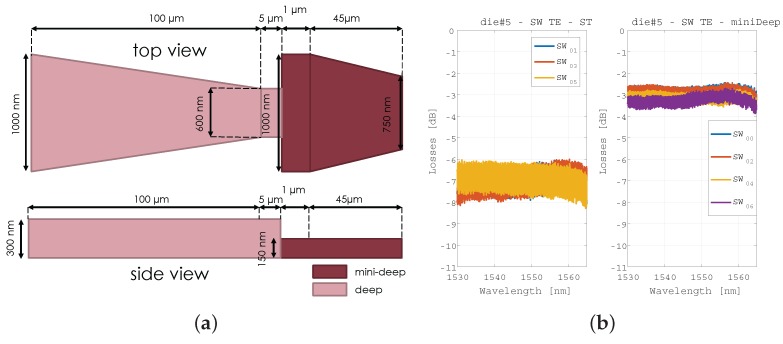
(**a**) Sketch and dimensions for the inverted tapers using two deeply etched cross-sections, (**b**) regular tapers performance and inverted tapers performance (left and right respectively).

With the aim of enforcing the truly broadband nature of our platform, we resorted to edge in/out coupling building block developments, despite grating couplers are also feasible in the technology. Hence, two different types of structures were designed, fabricated and tested. Firstly, regular tapers in a deeply-etched cross-section of 300 nm height and 3.5 μm width, secondly, inverted tapers using a combination of deeply-etched cross-sections of 300 and 150 nm, as shown in Figure 9a. Several test straight waveguides were measured using a broadband source ASE source and an OSA. The results are shown in Figure 9b and for regular and inverted tapers (left and right respectively). The plots present the optical transmission through the chip, normalized to that of the setup (lensed fibers directly faced). Hence, the plots include the loss for the in and out couplers. Therefore, regular tapers have approximately 3.5 dB of insertion loss per fiber point, whereas the inverted tapers have 1.5 dB of insertion loss.

### 3.7. Fabrication Process Steps Variations

The implications of different variations in the processing steps were investigated as well. The variations consisted on wafers with different combinations of buried oxide height, oxidation of the Si${}_{3}$N${}_{4}$ waveguides after etching and rapid thermal annealing (RTA) of the cladding oxide after deposition. A summary of the combinations is given in Table 3.

**Figure 10:**
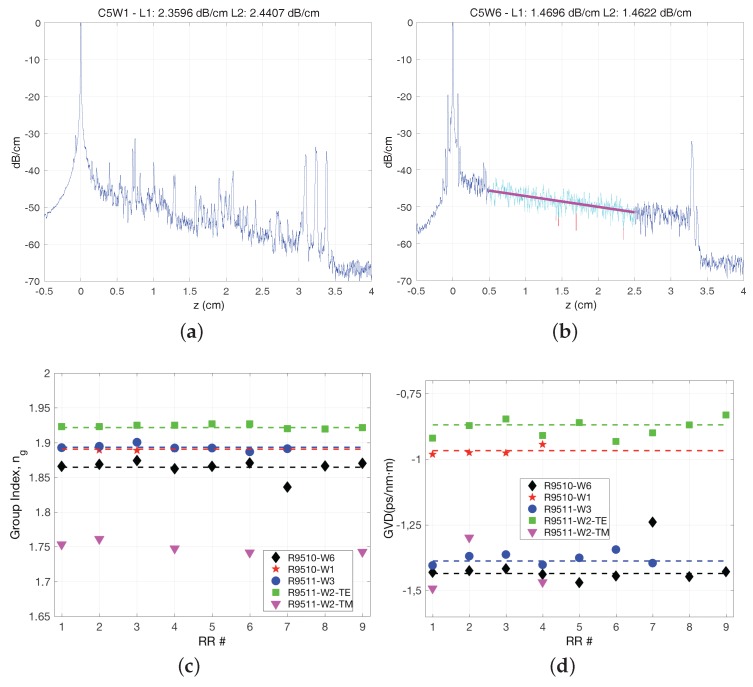
Impact on process steps on the linear propagation characteristics: OFDR measurement for spiral waveguides without (**a**) and with (**b**) oxidination of the Si${}_{3}$N${}_{4}$ waveguide after etching; group index (**c**) and GVD (**d**) for the different processing steps implemented.

The results are presented in Figure 10. The influence of oxidation in the propagation loss is comparatively presented in Figure 10a,b. Both show the OFDR measurement of spiral waveguide test structures, as the described above, for a wafer not subject to oxidation (a) and other where oxidation was applied after etching the waveguides (b). Two effects can be clearly appreciated. Firstly, the trace in (a) shows intensity peaks along the spiral (i.e., most relevant between z = 0.5 and z = 2.5 cm). The examination with a scanning electron microscope (SEM), revealed the waveguides had severe damage on the top edges and sidewalls, likely due to high energy centers caused by resist concentration points during etching. These peaks are removed with the oxidation, confirmed by SEM imaging of the sample for which the OFDR measurement is shown in (b). Secondly, the propagation loss difference between (a) and (b), obtained by comparing the fitted lines, is approximately 1 dB lower for the oxidized wafer.

The impact on the group index and dispersion is shown in Figure 10c,d respectively. Wafers R9510-W1 and R9511-W2, had different substrate height, 2.0 and 2.5 μm respectively (Table 3). A change in the cross-section dimensions is expected to alter the guiding properties of light, to be precise, increasing the substrate height should result in larger effective index, due to improved guiding conditions. By comparing the symbols for these two wafers, red stars and green squares in Figure 10c, corresponding to 2.0 and 2.5 μm respectively BOX height, an increase in the group index is appreciated. The group index is the addition of effective index and its first order dispersion. Both the effective index and its first order dispersion may be subject to changes due to the dimensions change. Our measurement method did not allow us, however, to gain further insight on the individual change of each.

Finally, the influence on the waveguide dispersion, that is, related to the second order derivative of the effective index, see appendix, measured from RR devices with the OFDR technique as previously shown, is presented in Figure 10d. Comparing once again R9510-W1 and R9511-W2 (red stars and green squares), a change in dispersion can be appreciated. However, in the same figure the impact of Si${}_{3}$N${}_{4}$ oxidation can be clearly observed, by comparing the traces corresponding to R9510-W1 and R9510-W6 (red stars vs. black diamonds), both having 2.0 μm substrate height, but only the latter is oxidized. The oxidation is at the expense of the Si${}_{3}$N${}_{4}$, which supposes and effective reduction of the waveguide dimensions. Hence, a change in the dimensions of the waveguide can be directly correlated with a change in the waveguide dispersion, as seen in the graph.

## 4. Prospects for Evolution

Si${}_{3}$N${}_{4}$ is transparent from visible to mid-infrared wavelengths (470–6700 nm) [61], however it is usually combined with SiO${}_{2}$ as substrate and cladding material. Up to the date, most of the applications have been restricted to wavelengths from VIS to the long NIR, cf. Table 1. Wavelengths in the long NIR and mid-infrared (MIR) are of high interest in applications such as trace gas analysis, chemical-biological sensing, environmental sensing, industrial process control, medical diagnostics, communications, defense and security and astronomy [62].

**Figure 11:**
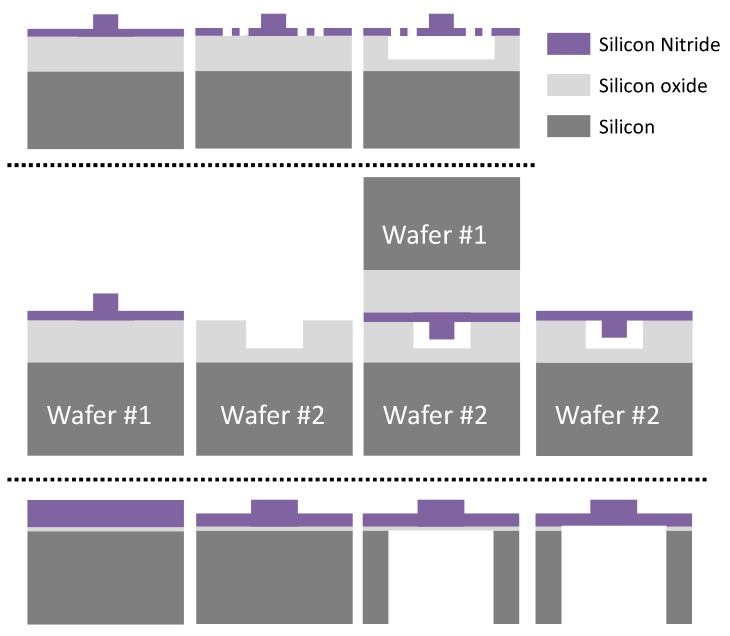
Processes to turn the Si${}_{3}$N${}_{4}$ platform into a membrane waveguide platform: through Si${}_{3}$N${}_{4}$ under-etch of the SiO${}_{2}$ (**top**); two wafer process using flip and bond (**middle**); two side etch process using a single wafer (**bottom)**.

Hence, exploiting in full the intrinsic Si${}_{3}$N${}_{4}$ transparency range, to encompass VIS, NIR and MIR wavelengths, would (broadly speaking) cover applications from bio-photonics, through tele/datacom up to sensing. Extending the operational wavelength range of Si${}_{3}$N${}_{4}$ waveguide platforms, requires the use of wafer layer stacks and waveguide cross-sections without (or with little) SiO${}_{2}$, since this material’s absorption is considerable for λ > 3.4 μm [63]. This might be accomplished in several ways, some of which are illustrated in Figure 11. In all the cases, the goal is to retain and reuse as many processing steps from the current platform, with minor modifications and additions, to result into a Si${}_{3}$N${}_{4}$ waveguide structure surrounded by air, i.e., a Si${}_{3}$N${}_{4}$ membrane. In all the cases, the membrane structure will have mechanical requirements (supporting structures), which are not discussed in the scope of this paper. Compared to silicon, Si${}_{3}$N${}_{4}$ has comparatively negligible two-photon absorption in the NIR, and despite it’s Kerr nonlinear coefficient is smaller [64], this has enabled Si${}_{3}$N${}_{4}$ as key platform for non-linear applications, such as supercontinuum and frequency comb generation [38,45]. Non-linear interaction can be enhanced by increasing the power confinement in the Si${}_{3}$N${}_{4}$ material, which would be the case in the aforementioned approach, in short, eliminating the SiO${}_{2}$ to create the Si${}_{3}$N${}_{4}$ membrane. Note that the index contrast of the Si${}_{3}$N${}_{4}$ membrane structure is larger than for the currently exiting SiO${}_{2}$/Si${}_{3}$N${}_{4}$/SiO${}_{2}$ platforms. Hence, confinement is expected to be larger, and the impact of side wall roughness on the propagation loss should be lower. Furthermore, loss reduction would be also favored by the use of the proposed rib membrane waveguides, rather than the strip waveguide platforms discussed in this paper. Last but not least, dispersion tailoring for the proposed Si${}_{3}$N${}_{4}$ membrane rib waveguide could be addressed by properly selecting the Si${}_{3}$N${}_{4}$ deposition techniques (refractive index tuning), etch depth and width as in [65].

The first approach, shown in the upper part of Figure 11, consists on the under etching of the substrate SiO${}_{2}$, such as in [66], where under-etching of silicon films to create pillar waveguides is proposed, or as in [46] for Si${}_{3}$N${}_{4}$ waveguides, in order to tailor the waveguide dispersion to attain supercontinuum generation in the VIS-NIR range. The proposed method in the figure is to add two process steps. Firstly, and after shallow waveguide etch step, holes should be defined in the Si${}_{3}$N${}_{4}$ layer. Secondly, SiO${}_{2}$ under-etch through these Si${}_{3}$N${}_{4}$ holes would be performed. Whereas the addition of these two process steps can be considered relatively simple and cost-effective, there is at least one potential drawback, related to the isotropic nature of the under-etching process. This would have implications on the design stage, since the amount of isotropic under-etching would be pattern dependent, that is, conditioned by the features designed and defined in the Si${}_{3}$N${}_{4}$ layer. Furthermore, how to address under-etching with densely integrated building blocks and circuits (e.g., an AWG) is an open concern as well. In conclusion, this technique would be pattern dependent, and as such, would require case by case analysis, which is not desirable from a production point of view.

A second alternative, shown in the middle part of Figure 11, is proposed after reference [67], where two silicon wafers are employed, one to etch air trenches in the silicon, and the other flip-bonded to the first, where the waveguides are defined. In this case, the SiO${}_{2}$ etch can be attained with dry etching techniques, therefore defining the air trenches very precisely, in comparison with the previous approach. Nonetheless, two wafers are required, plus a likely challenging alignment and bonding step in production. Hence, despite its advantage in terms of lithography, this technique may be considered comparatively less cost-effective.

As third alternative, we propose employing a single wafer, and one additional etch step from the backside of the wafer, as represented in the lower part of Figure 11. In this approach, the rear visual alignment (through silicon and nitride) of the mask to etch the silicon away can be considered well feasible, with alignment motives defined in the nitride guiding layer in the same shallow waveguide photolithography step. The SiO${}_{2}$ would then be used as etch stop, and removed by chemical means afterwards. Hence, there would be no need to subject the silicon wafer to a long thermal oxidation initial step, as in the current existing process.

## 5. Conclusions

In this paper, a review of the present state of the art for strip waveguide based silicon nitride photonics platform has been presented. The review has been complemented with modeling and experimental results for a versatile 300 m Si${}_{3}$N${}_{4}$ guiding film height platform, with canonical waveguide width of 1 μm. The choice of advanced full-field characterization techniques and suitable test structures, allowed to obtain the propagation loss of 1.4 dB/cm, group index of 1.9, birefringence of 0.168 and dispersion of −1.4 ps/nm m. Owing to the fact Si${}_{3}$N${}_{4}$ is a transparent material from the visible to the mid-infrared, means to upgrade the platform for broadband operation have been proposed, enabling the use of these platforms for a wide range of wavelengths and applications, such as biophotonics, tele/datacom, optical signal processing and sensing.

## Figures and Tables

**Table 1:** State of the art of silicon nitride strip waveguide platforms.

Group	Range	λ (nm)	Substrate	Core	Cladding	Confinement	Width (nm)	Height (nm)	Cut-off λ (nm) @ Width (nm)	Bend R (μm)	Straight (dB/cm) @ λ (nm)
Gent/Baets [25]	VIS	532	SiO${}_{2}$ (h = 2.0 μm) HDP-CVD	SiN PECVD	SiO${}_{2}$ (h = 2.0 μm)	Moderate	300 400 500	180	530 @ 532		7.00 @ 532 3.25 @ 532 2.25 @ 532
Aachen/Witzens [24]	VIS	660	SiO${}_{2}$/1.45 (h = ?)	SiN/1.87 PECVD	SiO${}_{2}$ (Water)	Moderate	700	100	580	35 (60)	0.51 @ 600 (0.71)
Gent/Baets [25]	VIS+	780	SiO${}_{2}$ (h = 2.4 μm) HDP-CVD	SiN PECVD 1.89@780	SiO${}_{2}$ (h = 2.0 μm)	Moderate	500 600 700	220	900 @ 780		2.25 @ 780 1.50 @ 780 1.30 @ 780
Gent/Baets [25]	VIS+	900	SiO${}_{2}$ (h = 2.4 μm) HDP-CVD	SiN PECVD	SiO${}_{2}$ (h = 2.0 μm)	Moderate	600 700 800	220	1100 @ 900		1.30 @ 900 0.90 @ 900 0.62 @ 900
IME/Lo [40]	NIR	1270–1580	SiO${}_{2}$ (h = 2.2 μm)	Si${}_{3}$N${}_{4}$ LPCVD	SiO${}_{2}$	Moderate	1000	400			0.32 @ 1270 1.30 @ 1550 0.40 @ 1580
IME/Lo [40]	NIR	1270–1580	SiO${}_{2}$ (h = 3.32 μm)	Si${}_{3}$N${}_{4}$ PECVD	SiO${}_{2}$	Moderate	1000	400			0.45 @ 1270 3.75 @ 1550 1.10 @ 1580
IME/Lo [40]	NIR	1270–1580	SiO${}_{2}$ (h = 3.32 μm)	Si${}_{3}$N${}_{4}$ PECVD	SiO${}_{2}$	Moderate	1000	600			0.24 @ 1270 3.50 @ 1550 0.80 @ 1580
Trento/Pavesi [19]	NIR	1550	SiO${}_{2}$ (h = 2.5 μm)	Multi-layer	Air/SiO${}_{2}$	Moderate					1.50 @ 1550 nm
Sandia/Sullivan [18]	NIR	1550	SiO${}_{2}$ (h = 5.0 μm)	Si${}_{3}$N${}_{4}$ LPCVD	SiO${}_{2}$ (h = 4.0 μm) PECVD or HDP	Moderate	800	150		500	0.11–1.45 @ 1550
Twente/Driesen [20]	NIR	1550	SiO${}_{2}$/1.45 (h = ?)	SiON PECVD	?	Moderate	2000–2500	140–190		25–50	0.20 @ 633 0.20 @ 1550
IME/Lo [21]	NIR	1550	SiO${}_{2}$ (h = 5.0 μm) PECVD	SiN/2.03 (h = 400nm) PECVD	SiO${}_{2}$ (h = 2.0 μm) PECVD	Moderate	700	400			2.1 @ 1550
LioniX-UCSB [22,23]	NIR	1550	SiO${}_{2}$/1.45 (h = 8.0 μm)	Si${}_{3}$N${}_{4}$ LPCVD	SiO${}_{2}$/1.45 (h = 7.5 μm)	Low	2800	100		500	0.09 @ 1550
LioniX-UCSB [22,23]	NIR	1550	SiO${}_{2}$/1.45 (h = 8.0 μm)	Si${}_{3}$N${}_{4}$ LPCVD	SiO${}_{2}$/1.45 (h = 7.5 μm)	Low	2800	80		2000	0.02 @ 1550
Cornell/Lipson [29]	NIR	1550	SiO${}_{2}$ (h = ?)	Si${}_{3}$N${}_{4}$ LPCVD	SiO${}_{2}$ (250 nm + 2 μm)	High	1800	910		115	0.04 @ 1550
LioniX [38]	NIR	1550	SiO${}_{2}$ (h = 8.0 μm)	Si${}_{3}$N${}_{4}$ LPCVD	SiO${}_{2}$ (h = 8.0 μm)	High	700-900	800 1000 1200			0.37 @ 1550 0.45 @ 1550 1.37 @ 1550
Toronto-IME/Poon [36]	NIR	1270–1580	SiO${}_{2}$ (h = 2.2 μm)	Si${}_{3}$N${}_{4}$ LPCVD	SiO${}_{2}$	Moderate	900	400			0.34 @ 1270 1.30 @ 1550 0.40 @ 1580
Toronto-IME/Poon [36]	NIR	1270–1580	SiO${}_{2}$ (h = 3.32 μm)	SixNy PECVD	SiO${}_{2}$	Moderate	1000	600			0.24 @ 1270 3.50 @ 1550 0.80 @ 1580
CNM-VLC	NIR	1550	SiO${}_{2}$ (h = 2.5 μm)	Si${}_{3}$N${}_{4}$ LPCVD	SiO${}_{2}$ (1.50 μm)	Moderate	1000	300		150	1.41 @ 1550
UCD/Yoo [31]	NIR	1550	SiO${}_{2}$ (h = ?)	Si${}_{3}$N${}_{4}$ LPCVD	SiO${}_{2}$ (h = 2.0 μm)	Moderate	2000	200		50	0.30 @ 1550
LigenTec [39]	NIR	1550	SiO${}_{2}$ (0.13–3.5 μm) Thermal	Si${}_{3}$N${}_{4}$ LPCVD	SiO${}_{2}$	High	2000	800		119	?
Chalmers/Torres [28]	NIR	1550	SiO${}_{2}$ (h = 2.0 μm)	Si rich SiNx LPCVD	SiO${}_{2}$ (h = 2.0 μm)	High	1650	700		20	1.00 @ 1550
Ghuagzhou/Shao [41]	NIR	1550–1600	SiO${}_{2}$ (h = 2.0 μm)	SixNy ICP-CVD	?	Moderate	1400	600		40	0.79 @ 1575
Columbia/Lipson [27]	NIR+	2300–3500	SiO${}_{2}$ (h = 4.5 μm)	Si${}_{3}$N${}_{4}$ LPCVD	SiO${}_{2}$ (500 nm + 2 μm)	High	2700	950	2500	230	0.60 @ 2600
MIT/Agarwal [37,42]	NIR+	2400–3700	SiO${}_{2}$/1.45 (h = 4 μm)	Si rich SiNx LPCVD	SiO${}_{2}$	High	4000	2500		200 @ 2650 200 @ 3700	0.16 @ 2650 2.10 @ 3700

**Table 2:** Summarized comparison of silicon nitride strip waveguide platforms.

Confinement	h (nm)	Range	Wavelength (nm)	Loss (dB/cm)
Low	80–100	NIR	1550	0.02–0.09
Moderate	150–400	NIR	1270–1600	0.11–1.45
High	400–1200	NIR	1550	0.04–1.37
High	950–2500	NIR+	2600–3700	0.16–2.10
Moderate	100–220	VIS+	532–900	0.51–2.25

**Table 3:** Runs and wafers with different processing steps: substrate height, waveguide oxidation and cladding rapid thermal annealing (RTA).

Run-Wafer	Substrate Height (μm)	Si${}_{3}$N${}_{4}$ Oxidation	Cladding RTA
R9510-W1	2.0	No	No
R9511-W2	2.5	No	No
R9510-W6	2.0	Yes	No
R9511-W3	2.5	Yes	Yes

## Appendix A. Refractive Index Models

Despite a large number of references exist in the literature dealing with the optical properties of Si${}_{3}$N${}_{4}$ and SiO${}_{2}$, only two provide information on a very wide wavelength range. The following equations describing the real part of the refractive index given by Lipson E.A. for Si${}_{3}$N${}_{4}$[27] (λ∈ [310, 5504] nm)and by Tan E.A. for SiO${}_{2}$[68] (λ∈ [0.21, 6.7] μm) were used in the simulations:

(A1) $$n_{Si_{3}N_{4}}\left(\lambda \left[nm\right]\right)=1+\frac{3.0249\lambda^{2}}{\lambda^{2}-135.3406^{2}}+\frac{40314\lambda^{2}}{\lambda^{2}-1239842^{2}}$$

(A2) $$n_{SiO_{2}}\left(\lambda \left[\mu m\right]\right)=1+\frac{0.69616663\lambda^{2}}{\lambda^{2}-0.0684043^{2}}+\frac{0.4079426\lambda^{2}}{\lambda^{2}-0.1162414^{2}}+\frac{0.8974794\lambda^{2}}{\lambda^{2}-9.896161^{2}}$$

Since Si${}_{3}$N${}_{4}$ is known to be transparent from the visible to the mid-infrared [61], no imaginary part was included in the simulations for the refractive index. Conversely, the imaginary part for SiO${}_{2}$ was taken into account according to the data provided by Kitamura R. [63]. In our case we employed a cubic spline interpolation of the data supplied in Table A1.

**Table A1:** Imaginary part for the refractive index of SiO${}_{2}$, after [63].

λ [μm]	0.4	0.6	0.8	1	1.25	1.5	1.7	1.9	2.1	2.3
$k_{SiO2}$	7.0 × 10${}^{-8}$	7.0 × 10${}^{-8}$	8.5 × 10${}^{-8}$	9.0 × 10${}^{-8}$	2.0 × 10${}^{-7}$	3.0 × 10${}^{-7}$	4.0 × 10${}^{-7}$	7.0 × 10${}^{-7}$	8.0 × 10${}^{-7}$	1.0 × 10${}^{-6}$
λ[μm]	2.5	2.75	2.85	3.0	3.1	3.15	3.3	3.5	3.75	-
$k_{SiO2}$	2.0 × 10${}^{-6}$	7.0 × 10${}^{-6}$	2.0 × 10${}^{-5}$	3.595 × 10${}^{-5}$	7.0 × 10${}^{-6}$	5.0 × 10${}^{-6}$	4.5 × 10${}^{-6}$	1.5 × 10${}^{-5}$	3.595 × 10${}^{-5}$	-

## Appendix B. Numerical Simulation

A rib waveguide structure was defined in the software tool OptoDesigner™ by PhoeniX Software b.v. [69]. The dimensions of the waveguide cross-section and the wavelength range were chosen in order to cover all the platforms summarized in Table 1. Each reported platform employs different substrated and cladding SiO${}_{2}$ heights. These height influence propagation parameters such as leakage loss, but since the growth of different heights of SiO${}_{2}$ is not a hard technical problem, other than using the right recipe and fabrication time, in the simulations a fixed height of 4.0 μm was used both for the substrate and cladding SiO${}_{2}$. The SiO${}_{2}$ wavelength dependent index was set according to Equation (A2) and Table A1. For the guiding layer, despite different platforms employ different fabrication methods (mainly PECVD and LPVCD), resulting into non-stoichiometrix (e.g., as the Si rich SiN${}_{x}$ of [28,37]) and stoichiometric (Si${}_{3}$N${}_{4}$), in the simulations we set the guiding layer to be Si${}_{3}$N${}_{4}$ grown by LPCVD as indicated by Luke e.a. in [27], and therefore we employed the index reported by them through ellipsometry measurements, given in Equation (A1).

The Si${}_{3}$N${}_{4}$ guiding layer height was one in the set of {80, 100, 150, 220, 300, 400, 600, 800, 1200} nm (9 points), the width was in the set of {2800, 2700, 2000, 1650, 1200, 1000, 900, 800, 700, 600, 400, 500, 300} nm (13 points) and the wavelength range covered in the simulations spans from $\lambda_{0}$ = 470 nm to $\lambda_{1}$ = 3700 nm, to be precise {470, 533, 630, 780, 850, 900, 1050, 1100, 1150, 1200, 1250, 1300, 1350, 1450, 1520, 1530, 1550, 1600, 1700, 1800, 1900, 2100, 2150, 2200, 2400, 2500, 2550, 2600, 2650, 2700, 2750, 2800, 2850, 2900, 2950, 3000, 3100, 3150, 3200, 3300, 3350, 3400, 3500, 3550, 3600, 3650, 3700} nm (47 points), amounting for a total of 5499 iterations. OptoDesigner 5.0.3 (PhoeniX Software bv, Enschede, The Netherlands) version was employed, in a machine with Linux Ubuntu 14.04.3 with Intel Core i5-4670@3.40 GHz (Intel Corp., Santa Clara, CA, USA) processors (4 cores) and 8 GB RAM memory. Each iteration took 30 seconds, to solve the TE and TM modes, and to store the effective indices and the field components found for the cross-section.

## Appendix C. Bernstein-Bézier Curve Fitting

### Appendix C.1. Bernstein Polynomials

Bernstain polynomials are given by:

(A3) $$B\left(t\right)=\sum_{i=0}^{n}b_{i,n}\left(t\right)P_{i}$$

where the basis functions are:

(A4) bi,n(t)=niti(1−t)n−i

When *t* is restricted to t∈[0,1], these polynomials are commonly recognized as the Bézier curves [70]. The first and second derivatives of Equations (A3) are given by:

(A5) $$B^{\prime }\left(t\right)=n\sum_{i=0}^{n-1}b_{i,n-1}\left(t\right)\left(P_{i+1}-P_{i}\right)$$

(A6) $$B^{\prime \prime }\left(t\right)=n\left(n-1\right)\sum_{i=0}^{n-2}b_{i,n-2}\left(t\right)\left(P_{i+2}-2P_{i+1}+P_{i}\right)$$

All the magnitudes (effective index, group index, dispersion, confinement and non-linear coefficient) obtained, were computed for combinations of Si${}_{3}$N${}_{4}$ height (*h*), waveguide width (*w*) and wavelength (λ). The fits performed using Bézier polynomials are for a given value of h, using as independent variables (w,λ). Since the Bézier polynomials independent variable is restricted to the interval [0, 1], the following normalized width and wavelength variables are defined:

(A7) $$W=\frac{w-w_{0}}{w_{1}-w_{0}}$$

(A8) $$\Lambda =\frac{\lambda -\lambda_{0}}{\lambda_{1}-\lambda_{0}}$$

where $w_{0}=300$ nm, $w_{1}=2800$ nm, $\lambda_{0}=470$ nm and $\lambda_{1}=3700$ nm.

### Appendix C.2. Effective Index, Group Index and Group Velocity Dispersion

For the effective index, a first fit is performed for a given height *h*, each wavelength Λ, with *W* as independent variable, using the equation:

(A9) $$n_{e}\left(W,\Lambda \right)=\sum_{i=0}^{n}b_{i,n}\left(W\right)E_{i}\left(\Lambda \right)$$

A second fit is performed for each of the obtained wavelength dependent coefficients, $E_{i}\left(\Lambda \right)$, employing a Bézier polynomial as well, that is:

(A10) $$E_{i}\left(\Lambda \right)=\sum_{j=0}^{m}b_{j,m}\left(\Lambda \right)E_{i,j}$$

Once the effective index has been fit, the same set of coefficients may be used to derive the group index and group velocity dispersion as:

(A11) $$n_{g}\left(W,\Lambda \right)=n_{e}\left(W,\Lambda \right)-\frac{1}{\Delta \lambda }\frac{\partial n_{e}\left(W,\Lambda \right)}{\partial \Lambda }$$

where $\Delta \lambda =\lambda_{1}-\lambda_{0}$. Hence:

(A12) $$\frac{\partial n_{e}\left(W,\Lambda \right)}{\partial \Lambda }=\sum_{i=0}^{n}b_{i,n}\left(W\right)\frac{\partial E_{i}\left(\Lambda \right)}{\partial \Lambda }$$

and consequently the coefficients for the group index are given by:

(A13) $$E_{i}^{\prime }\left(\Lambda \right)=\frac{\partial E_{i}\left(\Lambda \right)}{\partial \Lambda }=m\sum_{j=0}^{m}b_{j,m-1}\left(\Lambda \right)\left(E_{i,j+1}-E_{i,j}\right)$$

For the group velocity dispersion, using the second derivative of the effective index:

(A14) $$D\left(W,\Lambda \right)=-\frac{\lambda }{c}\frac{1}{\Delta \lambda^{2}}\frac{\partial^{2}n_{e}\left(W,\Lambda \right)}{\partial \Lambda^{2}}$$

This requires computing the second derivative of the Bézier polynomial for the wavelength fit:

(A15) $$\frac{\partial^{2}n_{e}\left(W,\Lambda \right)}{\partial^{2}\Lambda }=\sum_{i=0}^{n}b_{i,n}\left(W\right)\frac{\partial^{2}E_{i}\left(\Lambda \right)}{\partial^{2}\Lambda }$$

(A16) $$E_{i}^{\prime \prime }\left(\Lambda \right)=\frac{\partial^{2}E_{i}\left(\Lambda \right)}{\partial \Lambda^{2}}=m\left(m-1\right)\sum_{j=0}^{m}b_{j,m-2}\left(\Lambda \right)\left(E_{i,j+2}-2E_{i,j+1}+E_{i,j}\right)$$

### Appendix C.3. Confinement and Non-Linear Coefficient

**Table A2:** Effective index Bézier curve coefficients for h = 300 nm.

$E_{i,j}$	**h = 300 nm, TE**${}_{0}$					
**j**↓ / **i**→	**0**	**1**	**2**	**3**	**4**	**5**
0	1.8776213	2.122918	1.8338975	2.1035948	1.9577628	1.9983654
1	1.5934236	2.0444746	1.5628135	1.9956184	1.7710133	1.8322354
2	1.4362759	2.1394231	1.3769242	2.1079449	1.7048331	1.8222323
3	1.1819761	1.6413274	1.7166467	1.404391	1.7171903	1.6057694
4	1.8481478	0.40673147	3.3983956	0.50210963	2.1211686	1.6983453
5	1.0641421	2.472913	−0.55656357	3.0907673	0.83795738	1.5242143
6	1.6845798	0.71592265	2.9017514	0.43428066	2.0751221	1.5781534
7	1.2956966	1.786315	0.68464599	2.0493862	1.1950669	1.4802472
8	1.4635626	1.2874377	1.7004149	1.2467895	1.5764815	1.4858896
9	1.3965793	1.4399491	1.3459126	1.4839961	1.4166315	1.4509043
10	1.3994648	1.3942841	1.4113342	1.4091712	1.4289707	1.4336455
11	1.3863866	1.3867627	1.3895039	1.3997934	1.4072365	1.4142388
$E_{i,j}$	h = 300 nm, TM${}_{0}$					
j↓ / i→	0	1	2	3	4	5
0	1.8887401	2.0011959	1.8974288	1.9885233	1.9436535	1.9559063
1	1.609455	1.8264855	1.6515491	1.8019914	1.7310931	1.7505446
2	1.4640987	1.8261739	1.486507	1.802394	1.6385824	1.6865425
3	1.0822236	1.5366187	1.2886216	1.4596529	1.4286029	1.4308623
4	2.048853	0.63601297	2.8696145	0.84074835	1.9212071	1.6511395
5	0.82208993	2.5255386	−0.52650071	2.6367929	0.87419927	1.3632737
6	1.8738947	0.55886964	3.0985097	0.38808082	1.9833192	1.5423074
7	1.1896482	1.9091798	0.48595616	2.0705861	1.1322182	1.4061594
8	1.5039653	1.2294982	1.7920095	1.1714497	1.5558812	1.4495353
9	1.3849656	1.4550945	1.3147574	1.481189	1.3848654	1.4170576
10	1.3981807	1.3935368	1.4080525	1.396063	1.4087057	1.4083623
11	1.3810528	1.3913508	1.3786745	1.3942946	1.3896421	1.3937298

**Table A3:** $\gamma_{core}$ Bézier curve coefficients for h = 300 nm.

$g_{i,j}$	**h = 300 nm, TM**${}_{0}$					
**j↓ / i→**	**0**	**1**	**2**	**3**	**4**	**5**
0	3.8362107	2.2956705	−0.52159735	2.432606	0.13797359	0.68709093
1	−0.71449253	2.7699387	−2.6270367	2.2577456	−0.4980362	0.21254014
2	−1.1085944	5.5553406	−7.0130271	5.9631147	−1.8982644	0.34649937
3	1.4642427	−10.343148	18.209874	−15.298076	6.2120016	−0.40174322
4	−0.99087957	9.9119809	−18.880421	17.320314	−7.2764596	0.60477957
5	0.34561903	−6.9986065	14.116331	−13.41594	5.9589326	−0.43974132
6	0.064912041	3.7130759	−7.979316	7.990713	−3.6080734	0.30824375
7	−0.15910676	−1.4492067	3.351523	−3.5260698	1.6639435	−0.13072975
8	0.096807226	0.3972896	−1.0119507	1.1341267	−0.54227326	0.05266007
9	−0.031401585	−0.068990285	0.20015457	−0.23931782	0.12271787	−0.0081222481
10	0.0050752322	0.0060853095	−0.021668193	0.028655974	−0.013077666	0.0030896597
11	−0.00020783492	2.2365601 × 10${}^{-6}$	0.0002953014	−0.00035186824	0.0012013796	0.0010157015
$g_{i,j}$	h = 300 nm, TM${}_{0}$					
j↓ / i→	0	1	2	3	4	5
0	2.6208662	5.5999985	−5.4884	6.2828556	−1.3433512	0.7773674
1	2.781174	1.0400175	1.9201062	−0.073226442	1.0902226	0.54736235
2	−5.0616286	17.421036	−24.023399	19.786281	−7.0038343	0.84581743
3	4.6521823	−19.935111	39.006147	−32.76197	14.415252	−0.53697909
4	−3.3453677	11.697524	−24.935911	24.847549	−11.526451	0.87700597
5	2.0112132	−3.8689726	9.4648453	−10.411979	6.0538706	−0.19157833
6	−0.9336221	−0.62343138	−0.11605326	1.8652278	−1.629988	0.15450445
7	0.30708937	1.6378095	−2.5657901	1.4317783	−0.024543856	0.079989325
8	−0.061480214	−0.99477548	1.708361	−1.1694872	0.34468978	0.0032759916
9	0.004072112	0.32461521	−0.58086131	0.44066724	−0.11760206	0.031118024
10	0.00066290515	−0.051610124	0.092767063	−0.068004963	0.035175335	0.010901261
11	−0.00046545281	0.0026939267	−0.005011592	0.0057683831	0.0053385666	0.0083902458

A similar approach was followed for these two parameters and the expressions are analogue to Equations (A10)–(A13) above:

(A17) $$\Gamma \left(W,\Lambda \right)=\sum_{i=0}^{n}b_{i,n}\left(W\right)\sum_{j=0}^{m}b_{j,m}\left(\Lambda \right)G_{i,j}$$

(A18) $$\gamma \left(W,\Lambda \right)=\sum_{i=0}^{n}b_{i,n}\left(W\right)\sum_{j=0}^{m}b_{j,m}\left(\Lambda \right)g_{i,j}$$

(A19) $$\frac{\gamma (W,\Lambda )}{n^{2}n_{2}}=\sum_{i=0}^{n}b_{i,n}\left(W\right)\sum_{j=0}^{m}b_{j,m}\left(\Lambda \right)g_{i,j}$$
